# A meta-analysis of the effects of proton pump inhibitors on the risk of gastric cancer

**DOI:** 10.1097/MD.0000000000043053

**Published:** 2025-07-11

**Authors:** Hao Zhang, Xianyu Meng, Dongyun Gou, Xiaojing Liu, Yu Huang, Siyu Liu, Haiyang Wang, Hongyan Li

**Affiliations:** a Department of Abdominal Surgery, The Second People’s Hospital of Hengshui City, Hengshui City, Hebei Province, China; b Department of Gastrointestinal Surgery, Hebei Medical University Third Hospital, Shijiazhuang City, Hebei Province, China; c The Medical Record Room of the Medical Affairs Department, Hebei Medical University First Hospital, Shijiazhuang City, Hebei Province, China; d School of Mathematics and Computer Science, Hengshui University, Hengshui City, China; e Department of Vascular Surgery, Hebei Medical University Third Hospital, Shijiazhuang City, Hebei Province, China; f General Surgery Department, the Affiliated Hospital of Hebei University, Baoding City, Hebei Province, China.

**Keywords:** gastric cancer, meta-analysis, proton pump inhibitors, risk factors

## Abstract

**Background:**

Proton pump inhibitors (PPIs) are commonly prescribed drugs in clinical practice, mainly for the treatment of *Helicobacter pylori* infection and gastroesophageal reflux disease (GERD). With the application of PPIs, doctors have found a variety of adverse reactions related to it, with gastric cancer being the most serious. Our aim is to investigate whether the use of PPIs increases the probability of gastric cancer.

**Methods:**

By searching PubMed, EMBASE, the Cochrane Library and Web of science, references related to PPIs and gastric cancer were selected, and Newcastle-Ottawa scale (NOS) was used to evaluate the quality of the included references and analyze their bias. Then stataSE-64 was used for statistical analysis. The above processes were independently searched and evaluated by 2 researchers.

**Results:**

The use of PPIs significantly increased the incidence of gastric cancer (RR = 1.75, 95% CI: 1.48–2.07, *P* = .000).

**Conclusion:**

Long-term use of PPIs may increase the incidence of gastric cancer.

## 1. Introduction

Proton pump inhibitors (PPIs) are very common prescription drug in clinical practice, and has been widely used in the treatment of chronic gastritis, digestive tract ulcer, gastroesophageal reflux disease (GERD) and other diseases. With the wide application of PPIs such as omeprazole, more and more adverse reactions have been concerned. Common adverse reactions include pneumonia,^[1]^ fracture,^[2]^ intestinal infection,^[3]^ Clostridium difficile associated diarrhea,^[4]^ cerebrovascular events,^[5]^ chronic renal failure,^[6]^ dementia,^[7]^ gastrointestinal polyps and tumors,^[8]^ and an increased risk of all-cause mortality. At present, many studies have focused on whether long-term, high-dose PPIs can cause gastric cancer, but the results of these studies are quite different. Therefore, this paper conducted a systematic review of the existing research results to reach a more accurate and objective conclusion.

## 2. Methods

### 2.1. Research identification, inclusion criteria and exclusion criteria

We conducted a comprehensive literature search to compare the risk factors for gastric cancer in different populations using PPIs. We accessed PubMed, EMBASE, the Cochrane Library and Web of science, and searched all articles on gastric cancer and PPIs from August 2000 to August 2023, without language restrictions. The title, abstract and keywords contained the following terms: gastric cancer, Stomach Neoplasms, PPIs, randomized controlled trial, etc.

Inclusion criteria: the research type is raw data; control cases treated with and without PPIs; the content includes comparable clinical data; the result data are measurement data. Reviews, case reports or guidelines are excluded.

Exclusion criteria: animal and in vitro experiments, conference abstracts, reviews, doctoral papers, letters, case reports, systematic reviews, and meta-analyses; studies with low sample size (studies with <200 cases); cases from the same database of studies; incomplete data and unclear outcome effect.

### 2.2. Data extraction

In the data extraction work of this paper, 2 researchers extracted data separately, and discussed with superior physicians if they had any objections, and finally obtained a unified result.

### 2.3. Assessment of methodological quality

The quality of all studies in this meta-analysis was assessed using the Newcastle-Ottawa scale (NOS).^[9]^ Studies that achieve a score of 6 or more are considered high quality. Methods quality assessment was carried out independently by 2 researchers. Any differences were settled by consensus.

### 2.4. Statistical analysis

Statistical analysis was performed using STATASE-64 software. The bicategorical variables were expressed by relative risk (RR) and 95% confidence interval (95% CI). The heterogeneity among the results was observed. If there was no significant heterogeneity (*P* > .1, *I*^2^ < 50%) when using the fixed-effect model, if there is heterogeneity in the results (*P* < .1, *I*^2^ > 50%), using a random effects model. If the heterogeneity is too obvious, it is necessary to further analyze the causes and sources of heterogeneity and conduct subgroup analysis. At the same time, sensitivity analysis was carried out to explore the degree of influence of included studies on the overall effect size and the robustness of the results. If the results did not change significantly after excluding a certain study, the sensitivity was low and the results were more robust and reliable. On the contrary, if the difference between the results obtained after exclusion is large, it indicates that the sensitivity is high and the robustness of the results is low. Funnel plot and – test were used to quantify publication bias. Heterogeneity was tested by *P* < .10 was considered statistically significant and all others were extrapolated to *P* < .05 was considered statistically significant.

## 3. Results

### 3.1. Identification of eligible studies

**Figure 1.**
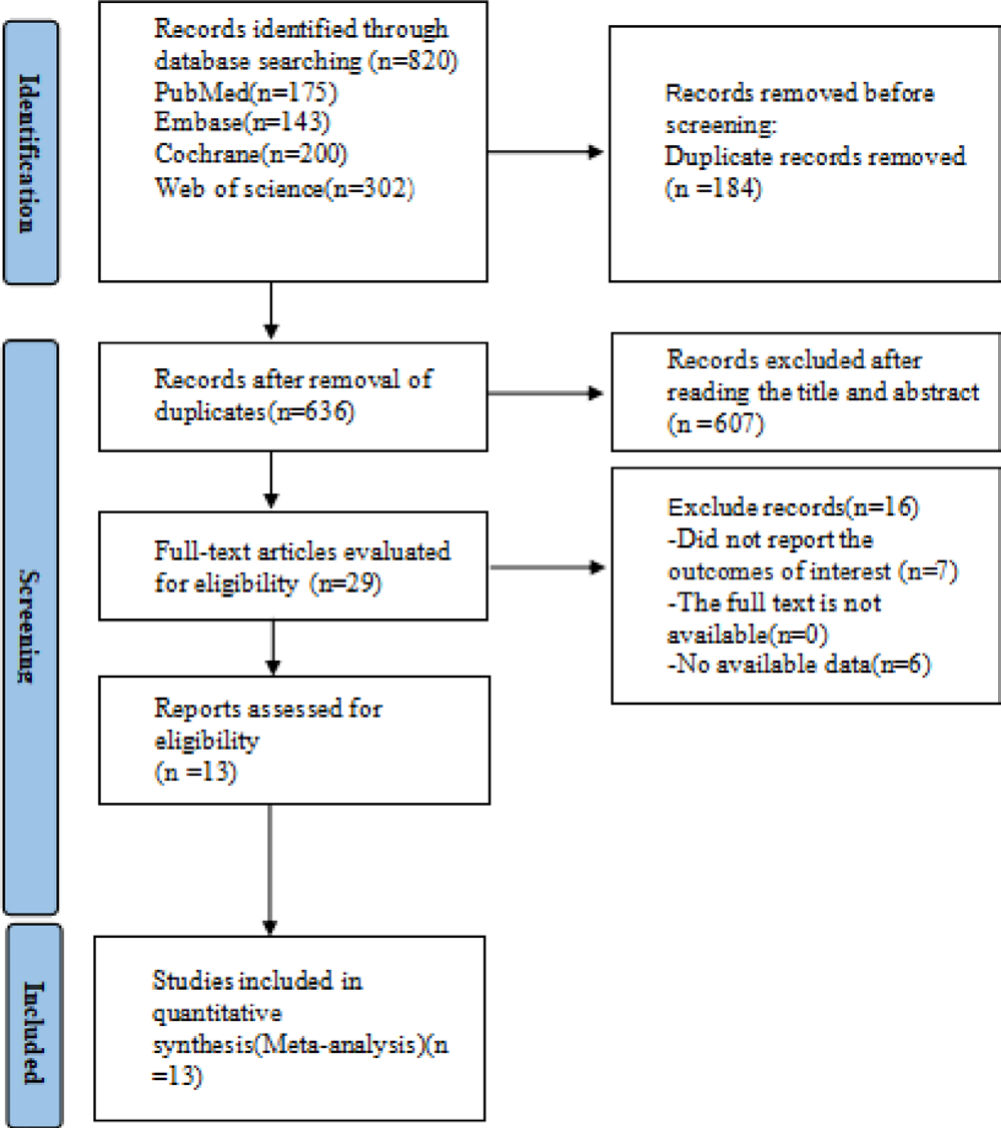
PRISMA flow diagram of the study process. PRISMA = Preferred Reporting Items for Systematic review and Meta-analysis.

A total of 820 literatures were obtained in literature search, and 13 literatures were finally included. Among them, 1 was a randomized controlled trial,^[10]^ 1 was a prospective cohort study,^[11]^ and 5 were retrospective cohort studies,^[12–16]^ 6 are case control studies.^[17–22]^ The basic information of the included literature is shown in Table 1. The retrieval procedure is shown in Figure 1.

**Table 1:** Characteristics of included studies.

Study	Year	Nation	Quality score	Design of study	Age	PPI		Non-PPI	
						Tevent	Tnoevent	Cevent	Cnoevent
Moayyedi	2019	Canada	7	RCT	59.5–75.8	86	8791	83	8807
Liu	2020	United Kingdom	7	Prospective cohort	NA	329	1119	1213	5394
Cheung	2018	China	8	Retrospective cohort	46.0–65.4	19	3271	134	60,126
Peng	2018	China	8	Case Control Study	NA	24	51,854	30	81,256
Poulsen	2009	Denmark	7	Retrospective cohort	40–84	24	51,854	519	2,345,905
Tamim	2008	Canada	7	Case Control Study	66.2–84.8	248	1598	1402	12,991
Lee	2020	United States	6	Case Control Study	58.3–83.2	164	1233	773	10,543
Rodri´guez	2006	United Kingdom	7	Case Control Study	40–84	43	442	464	9851
Duan	2008	United States	5	Case Control Study	NA	181	400	537	1674
Lai	2018	China	7	Case Control Study	20–84	13	118	11	453
Niikura	2018	Japan	6	Retrospective cohort	NA	13	24	105	547
Ng	2021	China	7	Retrospective cohort	52.7–75.7	60	100,000	17	100,000
Seo	2021	Korea	9	Retrospective cohort	15–94	118	11,741	40	11,741

### 3.2. Duration and dose of PPIs use

The median duration of PPIs use in Cheung et al’s study was 2.7 years, and the risk of gastric cancer increased with longer duration of PPIs use.^[12]^ In the study by Niikura et al, the median duration of PPIs use was 1.3 years.^[14]^ In the study by Ng et al, the median duration of PPIs use was 1314 days.^[15]^ In the study by Seo et al, the PPIs group used PPIs for at least 30 days, with a median usage time of 55 days.^[16]^ In Lee et al’s study, the PPIs group used PPIs for at least 2 years, and their research showed that the risk of gastric cancer did not increase with longer use of PPIs.^[19]^ Lai et al’s study showed that compared with those who had never used PPIs, the OR for gastric cancer in subjects with a cumulative PPIs use duration of ≤ 6 months was 1.59 (95% CI: 1.24 to 2.05), and the OR for gastric cancer in subjects with a cumulative PPIs use duration of > 6 months was 2.00 (95% CI: 1.36 to 2.95).^[22]^ In the study by Moayyedi et al^[10]^ and Liu et al,^[11]^ the dosage of PPIs used was 40 mg per dose. Other studies have neither mentioned the dosage of PPIs nor clearly specified the duration of PPI use.

### 3.3. PPIs and the risk of gastric cancer

**Figure 2.**
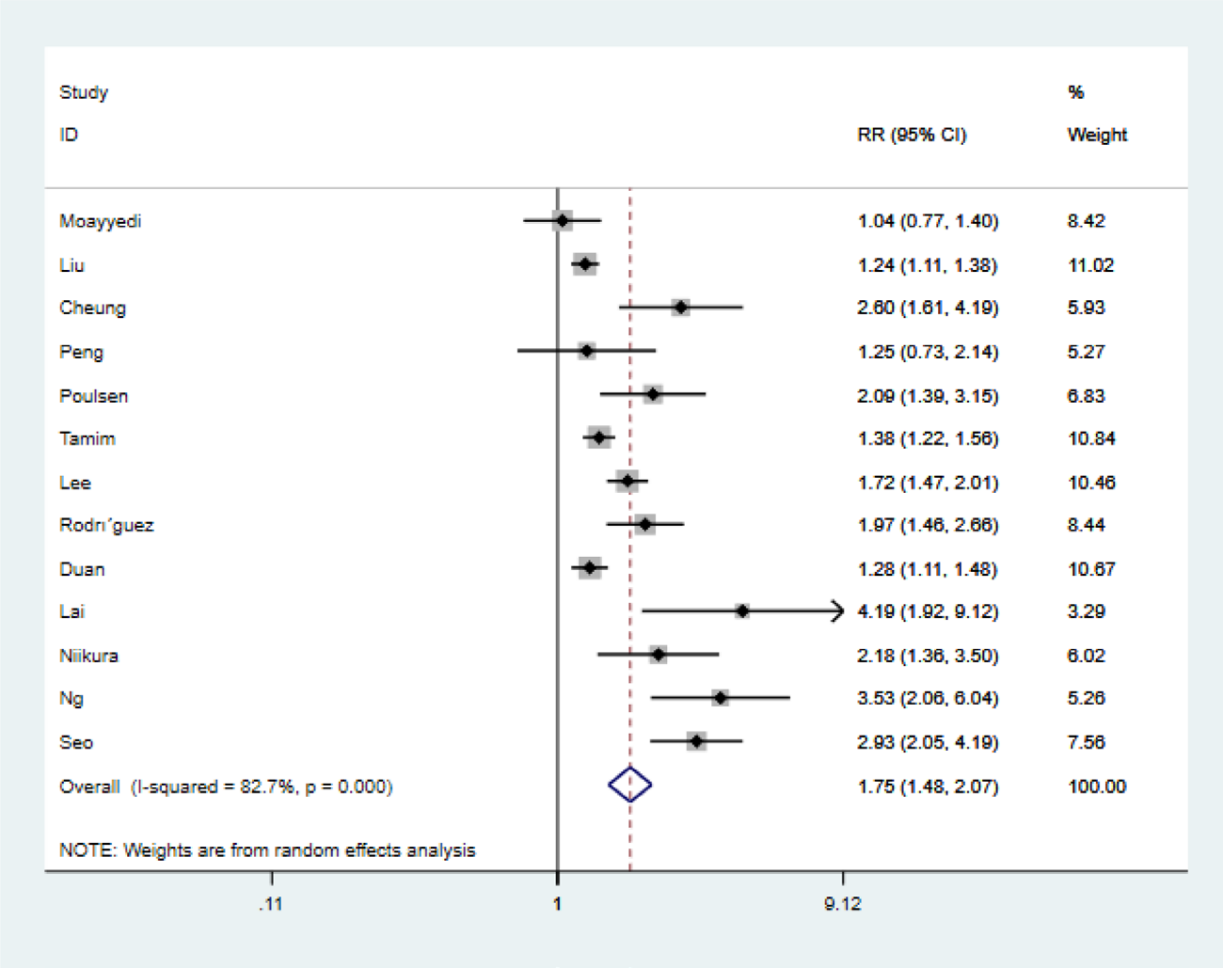
Forest plots.

A total of 13 original articles on PPIs and gastric cancer were included in this study. The statistical test showed that *I*^2^ = 82.7%, with large heterogeneity, so the random effects model was used for analysis. The results showed that PPIs use significantly increased the incidence of gastric cancer (RR = 1.75, 95% CI: 1.48–2.07, *P* = .000). The relationship between the use of PPIs and the occurrence of gastric cancer is shown in Figure 2.

### 3.4. Subgroup analysis

**Figure 3.**
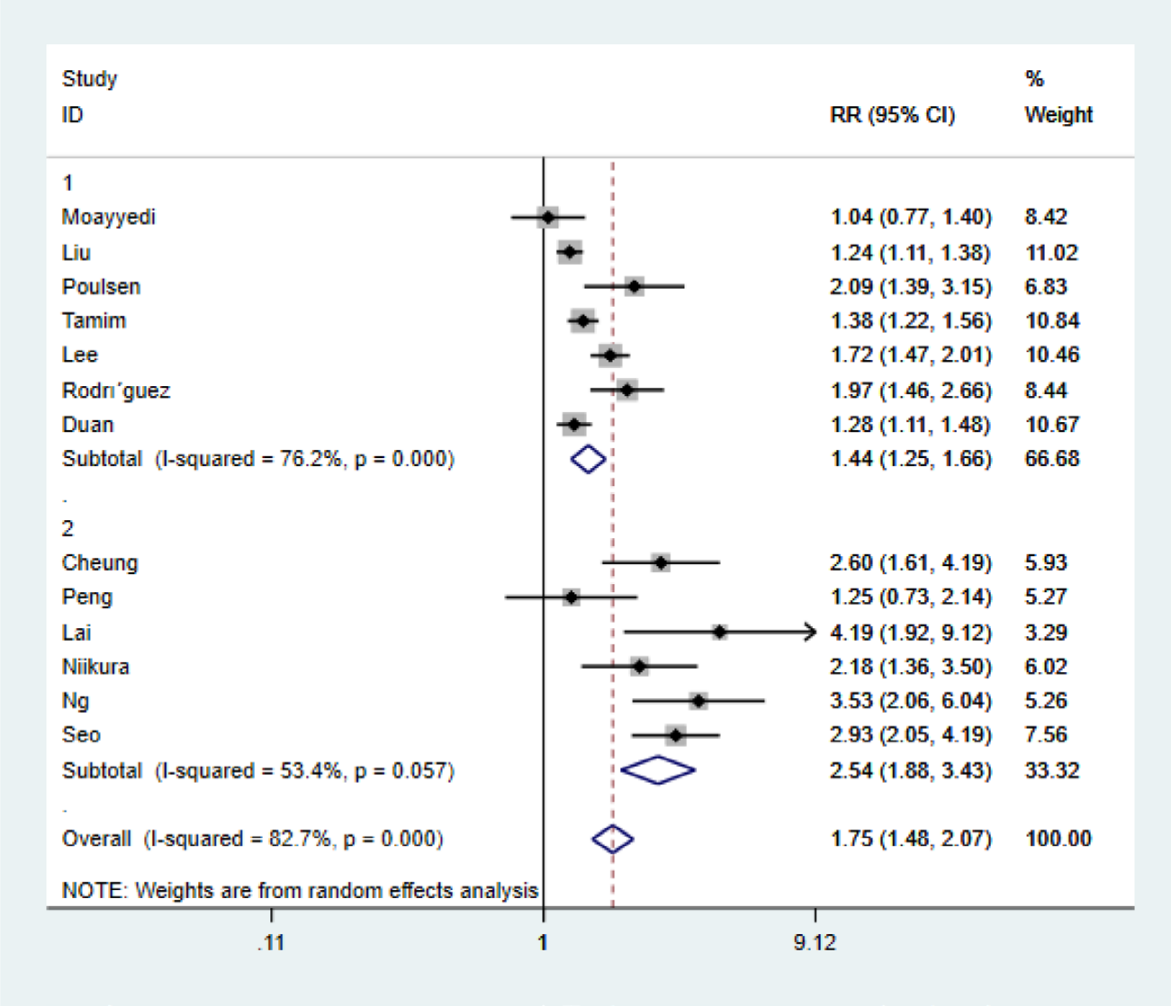
Subgroup analysis 1.

**Figure 4.**
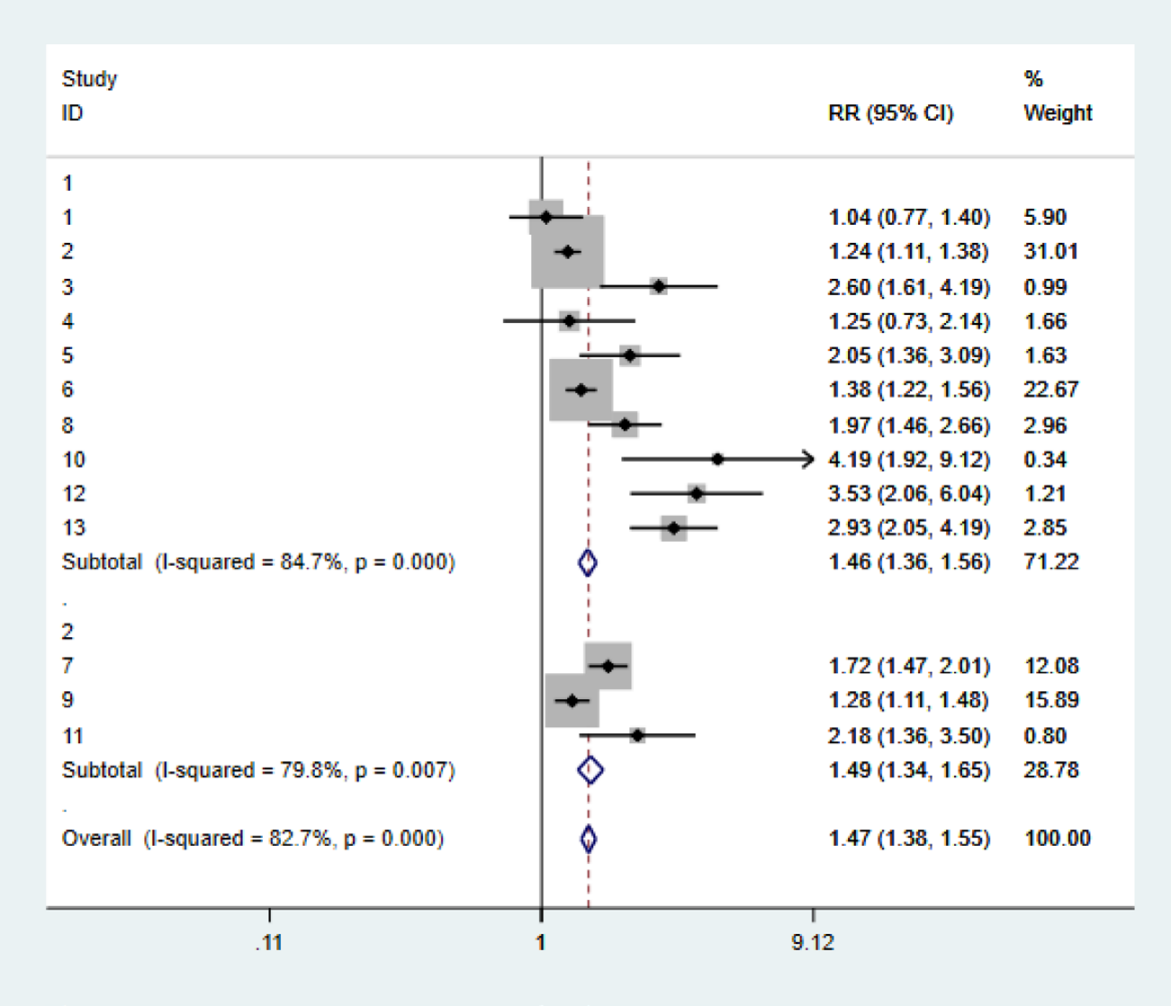
Subgroup analysis 2.

In this study, *I*^2^ = 82.7% was highly heterogeneous. In order to explore the source of heterogeneity, the author conducted subgroup analysis according to regions (Fig. 3) and literature quality (Fig. 4), and divided them into eastern subgroup and non-eastern subgroup according to patient origin. It was found that *I*^2^ in both groups decreased compared with the overall table. Considering that living habits and eating habits may be different from those in different regions, *P* = .057 > .05 was not statistically significant in the East Asian group. Subgroup analysis was conducted according to NOS scores of literature quality, and it was found that the heterogeneity of the 2 groups divided according to different NOS scores did not decrease significantly compared with before. According to the results of meta-analysis (*P* < .05) It was concluded that none of the subgroup analyses based on NOS evaluation of literature quality were the source of heterogeneity.

### 3.5. Sensitivity analysis

**Figure 5.**
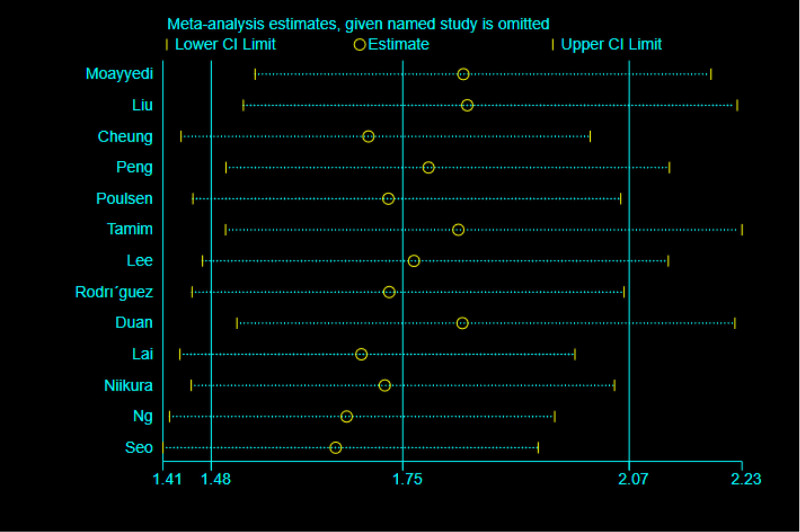
Sensitivity analysis.

Sensitivity analysis was performed on all included literatures after eliminating a single study one by one, and the results showed that the predictive values of all included literatures were within 95% CI, indicating that the results of this meta-analysis were not affected by any single study (Fig. 5). After analyzing and excluding each group of indicators of the included studies, it was found that the results had little change. The results are reliable.

### 3.6. Publication bias

**Figure 6.**
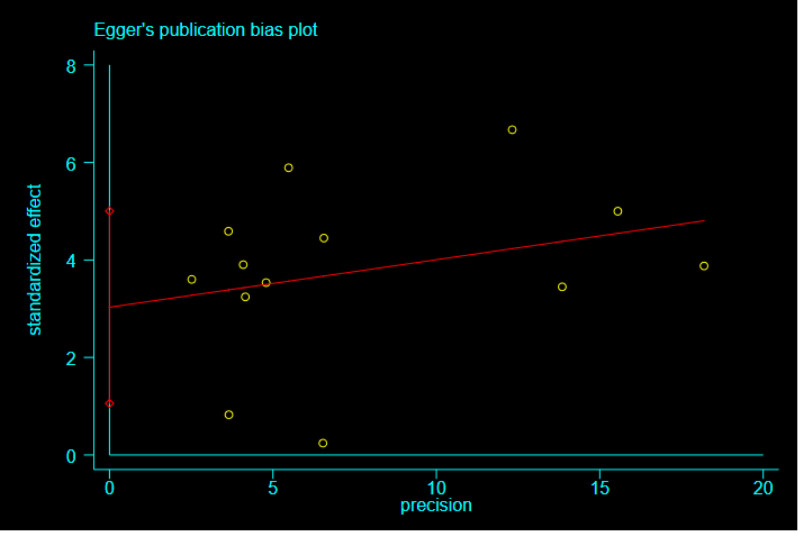
Egger plot.

According to the Egger plot drawn by StataSE-64 software (Fig. 6), it was found that the studies were evenly distributed on both sides of the funnel plot. Bias detection: *Pr*>| *z* | = .059 > .05, no publication bias.

## 4. Discussion

PPIs have been widely used in clinical practice, mainly for the treatment of *Helicobacter pylori* infection and GERD, and the related side effects have been gradually discovered, among which the most serious side effect is gastric cancer. In this paper, 4 English databases including PubMed were collected for meta-analysis of articles on the risk of gastric cancer and long-term use of PPIs from August 2000 to August 2023, and a total of 13 articles were included. Meta-analysis showed that PPIs use were significantly associated with gastric cancer (RR = 1.75, 95% CI: 1.48–2.07, *P* < .05). In order to explore the sources of heterogeneity, the authors conducted a subgroup analysis of the 13 included literatures according to their regions and literature quality scores: It was found that after grouping by region, the *I*^2^ of both the East Asian and non-East Asian groups decreased compared with the general table, which may be due to the differences in living habits and eating habits in different regions, but the *P* value of the East Asian group was .057 > .05, which was not statistically significant.

### 4.1. PPIs and *H pylori*

PPIs mainly act on H^+^/K^+^-ATPase on gastric parietal cells, and inhibit its function to reduce gastric acid secretion and relieve patients’ symptoms. A study by Tadashi et al showed that long-term use of PPIs worsened the condition of atrophic gastritis in patients with *H pylori* infection.^[23]^ *H pylori* is an anaerobic bacterium that can use its motility and chemotaxis function, urease production to adapt to the acidic environment and thus colonize the human stomach. *H pylori* will continue to stimulate the stomach to produce chronic inflammation, damage to the gastric mucosa, while long-term chronic inflammation will repeatedly stimulate the gastrointestinal mucosal epithelial cell overgrowth. Gastric cancer is thought to be an inflammation-induced tumor model, so inflammation undoubtedly increases the risk of gastric cancer.^[24]^ In addition, some *H pylori* carry the Cagl gene, which promotes intracellular translocation of the caga protein target and further interaction with cytoplasmic SHP-2, SHP-2 is an oncogenic protein and increases the level of inflammation in stomach tissue.^[25,26]^ *H pylori* can produce ammonia to neutralize stomach acid, which favors the growth of bacteria that break down nitrates. These bacteria produce large amounts of carcinogens such as nitrosamines, which also greatly increases the risk of stomach cancer in *H pylori* patients.^[24]^ The authors concluded that long-term use of PPIs increases the risk of gastric cancer by exacerbating *H pylori*-mediated infections.

### 4.2. PPIs and hypergastrinemia

Studies have confirmed that^[27]^ long-term use of PPIs can lead to hyperplasia and hypertrophy of parietal cells in the acid-secreting mucosa, protrusion into the gastric cavity, causing pseudohypertrophy and cell proliferation of gastric parietal cells, and inducing hypergastrinemia. In addition, in some patients with long-term use of PPIs, the serum level of chromoglin A16 increases, which can also directly lead to the proliferation of enterochromaffin like cells in the gastric mucosa (ECL). At high levels of gastrin, ECL cells are prone to malignant transformation and the formation of neuroendocrine tumors.^[27]^ However, despite studies suggesting that the association of gastric adenocarcinoma with ECL cell proliferation is less pronounced than that of gastric neuroendocrine tumors, a small proportion of gastric adenocarcinomas are still thought to originate from ECL cells.^[28]^ For example, diffuse gastric cancer expresses more ECL markers compared to intestinal gastric cancer, and gastric signet-ring cell carcinoma also expresses ECL cell markers in large amounts.^[29,30]^ Gastrin is an important gastrointestinal hormone mainly secreted by G cells in the antrum of the stomach. Gastrin exerts its biological effects mainly by binding to cholecystokinin type 2 receptor (CCK2 R). In addition, gastrin has trophic effect on gastric mucosal epithelial cells, and can promote the proliferation and differentiation of ECL cells in the stomach, proximal small intestine, and gastric parietal cells in the transitional zone. Studies have shown that^[31,32]^ gastrin can also act as a multipotent activator of transcription and mediate the activation of genes related to cell division, invasion, angiogenesis and anti-apoptosis, which plays an important role in the acquisition of malignant differentiation potential of cells. Maddalo et al^[33]^ has confirmed that there is a certain degree of hypergastrinemia in patients with gastric cancer, which has a certain impact on the growth and malignant transformation of cancer cells. Therefore, the authors believe that long-term use of PPIs can lead to an increase in gastrin, the formation of hypergastrinemia, and mediate the development of gastric cancer or gastric neuroendocrine tumors via the gastrin-ECL cell axis.

This meta-analysis also has some limitations. Although subgroup analysis was performed on the included literature, no reason for heterogeneity was found, and the analysis may be related to the following factors: age, gender, environmental factors, dietary habits, and other risk factors such as smoking. In addition, the data on the influencing factors of primary diseases on gastric cancer were not collected in this meta-analysis, considering that *H pylori* infection and GERD may also lead to the incidence of gastric cancer. Third, this study did not collect data on the measurement and duration of PPIs use, which may also be a risk factor for gastric cancer.

### 4.3. Other risk factors that may lead to gastric cancer

The papers included in this study not only show that PPIs increase the risk of gastric cancer, but also that H2 receptor blockers, advanced age, smoking, alcohol consumption, male gender, overweight, *H pylori* infection, dietary and lifestyle factors, related comorbidities (including peptic ulcers and chronic atrophic gastritis), and other drug use (such as aspirin and statins) are also potential risk factors for gastric cancer.^[10–22]^

Tobacco contains harmful chemicals such as nicotine and benzene, which directly damage gastric mucosal cells and cause chronic inflammation. Additionally, smoking causes the body to produce a large number of free radicals, which attack DNA and cause catastrophic damage, leading to genetic mutations. Therefore, it can be concluded that smoking causes mutations by damaging DNA through the production of free radicals, while gastric mucosal cells are damaged and proliferate under the stimulation of chronic inflammation. These genetic mutations are then passed on to daughter cells. As these mutations accumulate over time, they ultimately lead to gastric cancer.^[34]^ H2 receptor antagonists such as ranitidine also increase the risk of gastric cancer, which may be related to certain common mechanisms of action between H2 receptor antagonists and PPIs.

Dietary factors are also associated with gastric cancer. Nitrites in high-salt foods can be converted into carcinogenic nitrosamines under the influence of stomach acid; smoking or frying foods produces carcinogenic substances such as polycyclic aromatic hydrocarbons and benzo[a]pyrene during the smoking and frying processes. long-term consumption of these substances significantly increases the risk of stomach cancer; mouldy foods contain strong carcinogens such as aflatoxins, which can cause irreversible damage to the stomach lining even in small amounts. *H pylori* can colonize the surface of the gastric mucosa, secreting urease and other substances that destroy the gastric mucosal barrier and cause chronic inflammation. In addition, patients infected with *H pylori* are more likely to produce nitrites under the action of gastric acid. Long-term stimulation of the gastric mucosa by nitrites can cause cancer.^[35]^

The gastric mucosa of elderly people undergoes atrophy, and its repair capacity decreases, making it more susceptible to damage from various carcinogenic factors. Additionally, as people age, their immune system function declines, reducing their ability to identify and eliminate cancerous cells. Therefore, elderly individuals are considered a potential risk factor for gastric cancer. In addition, men are also at risk for stomach cancer, which may be related to the fact that men are more susceptible to unhealthy lifestyle habits such as smoking and drinking. At the same time, estrogen has a protective effect on the gastric mucosa, and men have lower levels of estrogen than women, so it can be assumed that men are at greater risk of stomach cancer than women.

## 5. Conclusions and prospects

After reading the 13 articles included in the meta-analysis, it was found that there was a significant relationship between the use of PPIs and the occurrence of gastric cancer, with RR = 1.75, 95% CI: 1.48–2.07, *P* < .05, which was statistically significant. Long-term use of PPIs for the treatment of *H pylori* or GERD is associated with an increased risk of gastric cancer development. Therefore, in the treatment of patients with *H pylori* infection and GERD, doctors should be cautious in giving patients long-term PPIs prescription, weigh the advantage and disadvantage of long-term use of PPIs, and stop PPIs use in time after the remission of the primary disease.

Through this meta-analysis, we found that long-term use of PPIs can increase risk of gastric cancer. We cannot deny the efficacy of PPIs, but the adverse reactions of long-term use of PPIs cannot be ignored. Considering the widespread and long-term use of PPIs and the large number of patients who require long-term PPIs maintenance therapy, further prospective, randomized controlled trials with large samples are needed to clarify the association between long-term PPIs use and the risk of gastric cancer. It is hoped that in the future, pharmacists can develop more effective and less side effects of acid-suppressive drugs, which can cure patients’ diseases and have higher safety.

## Acknowledgments

We would like to thank the researchers and study participants for their contributions.

## Author contributions

**Data curation:** Dongyun Gou, Haiyang Wang.

**Methodology:** Hao Zhang, Xianyu Meng, Yu Huang, Hongyan Li.

**Project administration:** Hongyan Li.

**Writing – original draft:** Hao Zhang, Dongyun Gou.

**Writing – review & editing:** Xianyu Meng, Xiaojing Liu, Yu Huang, Siyu Liu.
